# Targeting Misfolded Proteins to Fight Neurodegenerative Diseases

**DOI:** 10.1371/journal.pbio.1000290

**Published:** 2010-01-19

**Authors:** Kira Heller

**Affiliations:** Freelance Science Writer, Oakland, California, United States of America

**Figure pbio-1000290-g001:**
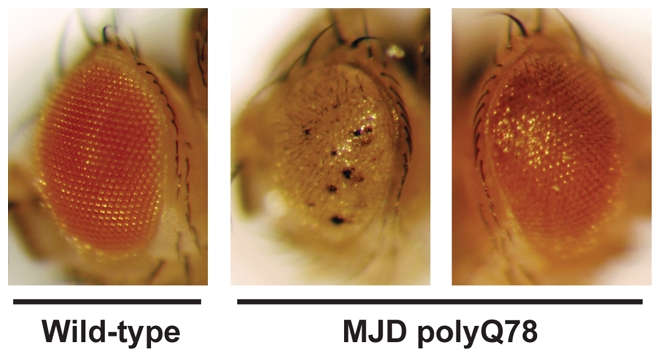
Fruit flies expressing a mutant form of the misfolded human MJD protein (middle) exhibit abnormal eye pigmentation and eye size. **Feeding the flies HSF1A (right) causes the eye to resemble wild-type eyes (left)**.

[Fig pbio-1000290-g001]With the assistance of proteins called chaperones, newly translated proteins fold into the three-dimensional shapes that are critical to their function. When proteins fold abnormally or become unstable, the consequences can be dire for cells, particularly neurons, which are exquisitely sensitive to the accumulation of misfolded proteins. Many neurodegenerative diseases, like Parkinson's and Creutzfeldt–Jakob, are associated with an accumulation of misfolded proteins. Huntington's disease (HD), an incurable condition that causes progressive loss of neurological function, is one of several hereditary neurodegenerative diseases known as poly-glutamine (polyQ) diseases that result from expansions of CAG (the trinucleotide repeat that codes for glutamine) in the protein-coding region of a gene (in HD, the gene is *huntingtin*). Proteins with these abnormally long tracts of glutamines are highly susceptible to misfolding and aggregation. Although it was first thought that aggregates of polyQ proteins caused neurotoxicity in polyQ diseases, recent studies have suggested that in fact the soluble misfolded protein precursors are the neurotoxins. Based on increasing evidence that misfolded proteins are the culprit in many neurogenerative diseases, Daniel Neef, Michelle Turski, and Dennis Thiele (this issue of *PLoS Biology*) reasoned that therapies targeting proteins that regulate folding may prove promising.

There are currently no cures for any of the polyQ diseases, but researchers hypothesize that if the abnormal polyQ proteins could be stabilized into their correct conformation, the neurotoxicity associated with protein misfolding in neuronal tissues might be prevented, leading to arrest of the disease process. Previous studies in cell culture and animal models of neurodegenerative disease found that increasing the amount of available protein chaperones, such as Hsp70 and Hsp40, can suppress protein aggregation and prevent polyQ disease–related damage to neurons.

Heat Shock Transcription Factor 1 (HSF1) is a “master regulator” of the protein chaperone response. HSF1 is inactive most of the time. But under conditions that are likely to put stress on proteins and cause them to misfold—such as preexisting misfolded proteins, oxidation, or heat—HSF1 is activated and directs the production of multiple protein chaperones. In neurons, HSF1 does not appear to be very efficiently converted to its active form, making these cells particularly likely to accumulate large amounts of misfolded proteins, and thus more susceptible to damage than other cell types.

Because of its role as a master regulator of the protein stress response, HSF1 activation is an attractive target for researchers looking for new therapies to treat diseases associated with protein misfolding. Although previous screens identified compounds that activate HSF1, they often did so in ways that were ultimately detrimental to cells, such as by causing increased amounts of misfolded proteins to accumulate, or by inhibiting Hsp90, a protein chaperone that has critical roles in cell growth, signaling, and proliferation. Thus, when Neef, Turski, and Thiele set out to identify small molecules that would activate human HSF1 from a library of over 10,000 compounds, they designed their screen to be insensitive to compounds that promoted proteotoxicity or inhibited Hsp90. The screen used a strain of the yeast *Saccharomyces cerevisiae* that had been engineered to express human HSF1 and would grow only when this introduced protein was activated by the presence of a successful candidate compound. Using this screen, the researchers were able to quickly identify 33 compounds that allowed activated HSF1-dependent yeast growth to occur. They then focused on the compound (which they called HSF1A) that caused the most robust HSF1-dependent yeast growth.

To find out if HSF1A could initiate an HSF1 response in mammalian neurons, the researchers added it to an in vitro culture of rat neuronal precursor cells that expressed a mutant human *huntingtin* gene containing an expanded tract of glutamines. Hsp70 expression was increased in these cells, and less misfolded huntingtin protein accumulated, compared to cells to which HSF1A was not added.

Turning next to an in vivo animal model, the researchers employed fruit flies that expressed a mutant form of human MJD/SCA3 gene (associated with the polyQ disease Machado-Joseph). In the experimental system, expression of this gene was limited to the fruit flies' eyes, where accumulation of the mutant protein caused loss of pigmentation and smaller eyes. When these flies were fed HSF1A, their progeny had eyes that looked similar to wild-type flies, indicating that HSF1A was partially suppressing the cell toxicity caused by polyQ protein accumulation.

After conducting further tests to determine how HSF1A was causing activation of HSF1, the researchers confirmed that HSF1A was not acting to increase protein misfolding and it was not inhibiting Hsp90. Instead, they found that HSF1A was interacting with the TRiC/CCT cytosolic chaperone complex, which helps to fold cytoskeletal proteins like tubulin and actin. Although TriC activity was not previously known to have a role in HSF1 activity, a recent study found that it inhibits polyQ protein aggregation and cytotoxicity in yeast and animal cells. Thus, HSF1A may be suppressing protein misfolding and aggregation by positively regulating TRiC activity.

Although HSF1A—as well as other activators of HSF1 that may be identified in the future by Neef, Turski, and Thiele's humanized yeast high-throughput screen—have potential for treating protein misfolding diseases such as HD or Parkinson's, the researchers caution that several questions still need to be answered. For example, how is HSF1A interacting with the TriC complex, and how might this affect HSF1 activation? And, what negative effects on cell metabolic processes might arise from therapeutic activation of the protein chaperone response? Studies are currently under way to address these issues and further explore HSF1's potential as a therapeutic target in protein misfolding–related neurodegenerative diseases.

**Neef DW, Tursk ML, Thiele DJ (2010) Modulation of Heat Shock Transcription Factor 1 as a Therapeutic Target for Small Molecule Intervention in Neurodegenerative Disease. doi:10.1371/journal.pbio.1000291**

